# Reversible lesion in the splenium of the corpus callosum in a patient with chronic alcoholism

**DOI:** 10.1002/jgf2.308

**Published:** 2020-04-18

**Authors:** Syuichi Tetsuka, Takeshi Kamimura, Gaku Ohki, Ritsuo Hashimoto

**Affiliations:** ^1^ Department of Neurology International University of Health and Welfare Hospital Nasushiobara Japan; ^2^ Department of Internal Medicine Yuki Hospital Yuki Japan

**Keywords:** chronic alcoholics, Marchiafava‐Bignami disease, reversible lesion, splenium of the corpus callosum

## Abstract

Marchiafava‐Bignami disease (MBD) is often diagnosed in chronic alcoholics. The disease processes typically involve the corpus callosum and clinically presents with various manifestations on the basis of clinical condition, extent of the splenium of the corpus callosum involvement at brain magnetic resonance imaging (MRI), and prognosis. We report a patient affected by MBD, who presented an isolated reversible splenial lesion at brain MRI and achieved a favorable recovery.

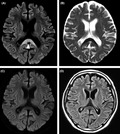

A 59‐year‐old man was admitted to our emergency room because of mild alteration of consciousness and motor deficit of the lower limbs. He had been suffering from chronic alcohol abuse for several years and lived in isolation. He did not have any other significant medical history. He was found in his house, on the floor of the living room. According to that reported by his brother, who found him, he was unable to stand, and his speech was disorganized. His initial vital signs were as follows: heart rate, 75 beats/min; blood pressure, 154/96 mm Hg; temperature, 35.2°C; and oxygen (O_2_) saturation, 99% in room air. On physical examination, he was noncomprehensive and showed a Glasgow Coma Scale score of 12 (E3V4M5). There were no meningeal signs, and his pupils exhibited a normal size and were reactive to light. Dysarthria was observed. According to manual muscle testing, the muscle strength values of the bilateral arms and bilateral legs were about grade 3/5 and grade 2/5, respectively. He was unable to sit or stand. The deep tendon reflexes of the limbs, including biceps, brachial, knee, and ankle jerk, were decreased. A pathological reflex (Babinski sign) was not detected. No seizures or limb tremor was noted. Laboratory analyses revealed blood glucose level of 135 mg/dL, AST level of 28 IU/L, and ALT level of 20 IU/L. Blood ammonia, serum creatinine, and electrolyte levels were within normal limits. Computed tomography (CT) did not show abnormalities. Brain magnetic resonance imaging (MRI) showed an area of high signal on diffusion‐weighted imaging (DWI), with a decreased apparent diffusion coefficient (ADC) observed in the splenium of the corpus callosum (SCC) (Figure 1A,B). We proposed a diagnosis of Marchiafava‐Bignami disease (MBD) based on the patient's history and findings on physical examination and MRI. He was treated with hydration and parenteral nutrition with a vitamin supplement while undergoing rehabilitation. He showed improvement of symptoms with a good recovery within 2 weeks and was able to walk and communicate normally through conversation. A follow‐up brain MRI performed 2 weeks later showed resolution of the abnormal SCC finding on DWI; moreover, fluid‐attenuated inversion recovery (FLAIR) imaging revealed no abnormal findings (Figure 1C,D).

**Figure 1:**
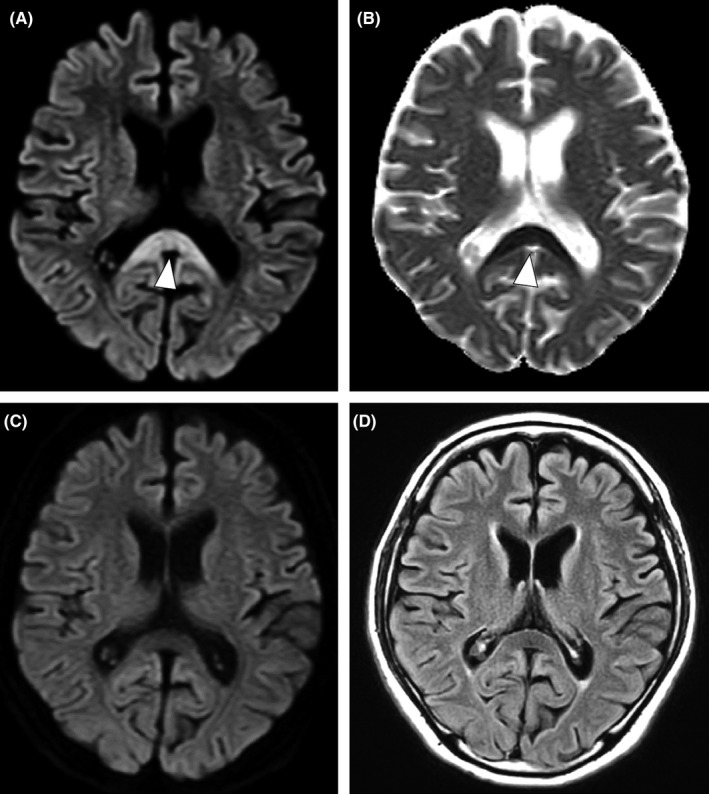
(A and B); MRI studies at onset. Diffusion‐weighted images (DWI) show hyperintensity involving the splenium of the corpus callosum (arrowhead) (A) with corresponding hypointensity on apparent diffusion coefficient (ADC) images (B). (C and D); MRI studies at two‐week follow‐up. DWI shows normal signal in the splenium of the corpus callosum (C). There is no abnormal lesion on fluid‐attenuated inversion recovery (FLAIR) (D)

Reversible lesion of the SCC is an MRI finding observed in a wide variety of diseases and conditions, including MBD.^1^ MBD is a rare disorder characterized by abnormal MRI findings in the SCC with various clinical manifestations, even in the absence of typical clinical syndromes.^2^ Although the exact pathology underlying the reversible lesion of the SCC remains unclear, recent reports suggest that cytokine‐mediated cytotoxic edema of the SCC is an important pathophysiological manifestation of this condition.^1^ There are differential diagnoses for patients with chronic alcoholism exhibiting a disturbance of consciousness (Table 1)^3^; however, clinicians should consider MBD in this setting. Establishment of a definitive diagnosis of MBD at an early stage is essential for these patients. This is because patients with MBD who were treated with thiamine within 2 weeks after the onset of symptoms had a significantly lower rate of poor outcomes, that is, even coma or death, than those who received delayed treatment, and this disease is reversible if treated timely with thiamine and vitamin B complex.^2^, ^4^ MBD diagnosis seems to have become relatively easy because of MRI findings, including DWI and ADC maps. Typically, DWI reveals hyperintensity in acute cases of lesion of the SCC, with corresponding hypointensity and decreased values on ADC mapping. In addition, MRI findings can be used as prognostic indicators because clinical recovery and neuroradiological changes exhibit a strong correlation, as observed for the present case. Among other imaging modalities, CT may show hypoattenuating signs in the corpus callosum and, in exceptional situations of hemorrhage, these regions turn into iso‐ or hyperattenuating signs.^5^ It is important to recognize the neuroradiological features of MBD, which is that DWI shows hyperintense regions with reduced diffusivity on ADC mapping in the SCC, to establish appropriate diagnosis. In the clinical setting such as an examination of chronic alcoholic patients, whenever brain MRI shows abnormalities in the SCC, clinicians should keep MBD in mind as the first differential diagnosis.

**Table 1:** Differential diagnoses for chronic alcoholic patient with impaired consciousness

Wernicke's encephalopathy
Pontine and extrapontine myelinolysis
*Marchiafava‐Bignami disease*
Withdrawal syndrome with seizures
Hypoglycemia
Hyponatremia
Hepatic encephalopathy
Dehydration
Malnutrition
Acute alcoholism
Traumatic cerebral hemorrhage lesion
Metabolic acidosis
Alcoholic ketoacidosis
Syncope due to arrhythmia in alcohol abuse

## CONFLICT OF INTEREST

The authors have stated explicitly that there are no conflict of interest in connection with this article.
